# Supplementary material for: DLK-dependent axonal mitochondrial fission drives degeneration after axotomy
Source: Nat Commun. 2024 Dec 30;15:10806. doi: 10.1038/s41467-024-54982-9 (PMC11686342; doi:10.1038/s41467-024-54982-9)
Supplement: Supplementary file 1 — Supplementary Information [file 41467_2024_54982_MOESM1_ESM.pdf]

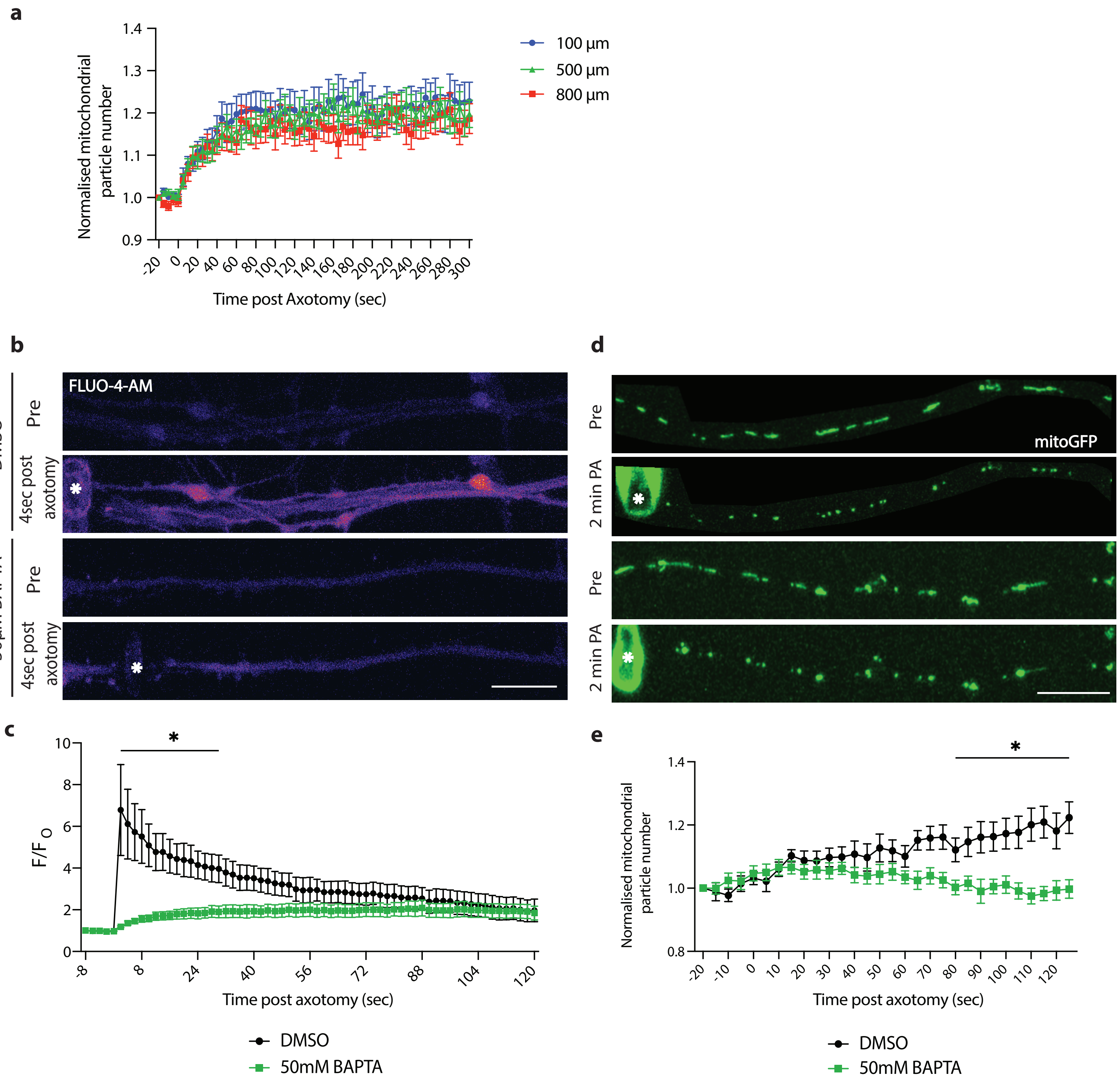
a
Normalised mitochondrial particle number
Time post Axotomy (sec)
100 µm
500 µm
800 µm
b
FLUO-4-AM
DMSO
Pre
4sec post axotomy
50µM BAPTA
Pre
4sec post axotomy
c
F/F O
Time post axotomy (sec)
DMSO
50mM BAPTA
d
Pre
mitoGFP
2 min PA
Pre
2 min PA
e
Normalised mitochondrial particle number
Time post axotomy (sec)
DMSO
50mM BAPTA

Sup Figure 1 – Axotomy leads to calcium flooding of the axon and mitochondrial fission is Ca dependent.

a) Normalized number of mitochondrial particles post axotomy in WT neurons axotomized 100µm (blue circle), 500µm (green triangle) and 800µm (red squares) from the soma. Results are represented as mean ± SEM. N=3 independent differentiations, N≥ 30 axotomized neurons.

b) Representative images of Fluo-4-AM calcium imaging of injured axons pre and 4 seconds post axotomy treated with DMSO and 50 µM BAPTA-AM. * Marks the site of axotomy. Scale bar = 25 µm.

c) Relative Fluo-4-AM fluorescence after axotomy in DMSO and BAPTA AM-treated neurons after injury. Results are represented as mean ± SEM. N=6 cells. Two-way ANOVA, Bonferroni correction (p≤0.05 *).

d) Representative images of WT neurons transduced with mitoGFP (green) treated with DMSO and BAPTA-AM pre and 2 mins post axotomy (PA). * Marks the site of axotomy. Scale bar = 25 µm.

e) Normalized number of mitochondrial particles post axotomy in DMSO (black) and BAPTA-AM (green)-treated neurons. Results are represented as mean ± SEM. N=2 independent differentiations, N≥ 16 axotomized neurons. Two-way ANOVA, Bonferroni correction (p≤0.05 *).

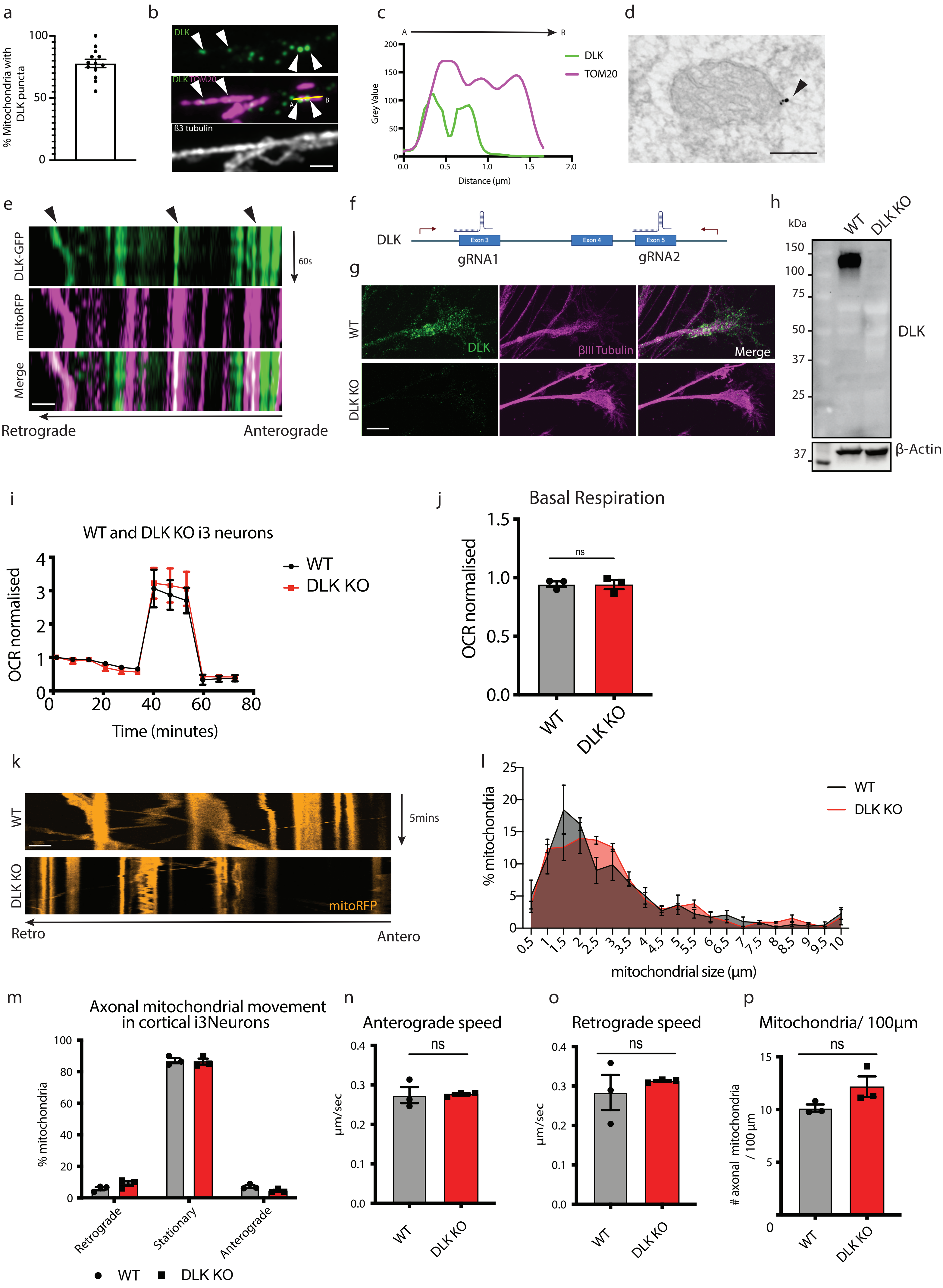

a
% Mitochondria with DLK puncta
b
DLK
DLK TOM20
β3 tubulin
c
A
B
Grey Value
Distance (µm)
DLK
TOM20
d
e
DLK-GFP
mitoRFP
Merge
60s
Retrograde
Anterograde
f
DLK
Exon 3
Exon 4
Exon 5
gRNA1
gRNA2
g
WT
DLK KO
DLK
βIII Tubulin
Merge
h
kDa
WT
DLK KO
DLK
β-Actin
i
WT and DLK KO i3 neurons
OCR normalised
Time (minutes)
WT
DLK KO
j
Basal Respiration
OCR normalised
ns
WT
DLK KO
k
WT
DLK KO
5mins
mitoRFP
Retro
Antero
l
% mitochondria
mitochondrial size (µm)
WT
DLK KO
m
Axonal mitochondrial movement in cortical i3Neurons
% mitochondria
Retrograde
Stationary
Anterograde
WT
DLK KO
n
Anterograde speed
µm/sec
ns
WT
DLK KO
o
Retrograde speed
µm/sec
ns
WT
DLK KO
p
Mitochondria/ 100µm
axonal mitochondria / 100 µm
ns
WT
DLK KO

Sup Figure 2 – DLK localizes to mitochondria in i3Neurons

- **a)** Quantification of percentage of axonal mitochondria containing DLK puncta. Results are represented as mean ± SEM. N=13 axons from 3 individual differentiations.
- **b)** Representative high-resolution images of WT neurons axons stained with anti-DLK (green) and anti-TOM20 (magenta). Scale bar = 5 µm.
- **c)** Line-scan analysis of relative fluorescence intensity from the dashed line shown in b.
- **d)** Immuno-EM of endogenous DLK localized in the mitochondria of i3Neurons. Arrow indicates DLK puncta. Scale bar = 5 µm.
- **e)** Representative kymographs of DLK-GFP (green) and mitoRFP (magenta) co-trafficking along the axon. Scale bar = 5 µm.
- **f)** Schematic representation of DLK knockout (KO) generation strategy. WT i^3 iPSCs were transfected with two gRNAs targeting DLK exon 3 and 5. Primers used for knockout validation are shown in red.
- **g)** Representative images of WT and DLK KO neurons stained with DLK (green), ßIII tubulin (magenta). Scale bar = 25 µm.
- **h)** Western blots of WT and DLK KO neurons. Immunoblot for DLK and loading control ß actin.
- **i)** Seahorse oxygen consumption rate (OCR) analysis of WT and DLK KO i3Neurons normalized to WT Neurons. Results are represented as mean ± SEM. N=3 independent differentiations, 3 wells measured per condition.
- **j)** Basal OCR levels of WT and DLK KO neurons normalized to UT i^3Neurons. Results are represented as mean ± SEM. N=3 independent differentiations, 3 wells measured per condition. No significant changes observed (ns).
- **k)** Representative kymographs of WT and DLK KO axon mitochondrial (mitoRFP orange) movement. Scale bar = 5 µm.
- **l)** Quantification of WT and DLK KO axon mitochondrial length. Results are represented as mean ± SEM. N=3 independent differentiations, N≥30 axons. No significant differences observed (ns).
- **m)** Quantification of percentage of stationary, retrograde and anterograde-moving mitochondria in WT and DLK KO neurons. N=3 independent differentiations, N≥ 30 axons. No significant differences observed (ns).
- **n)** Quantification of anterograde mitochondrial speed in WT and DLK KO neurons. N=3 independent differentiations, N≥ 30 axons. No significant differences observed (ns).
- **o)** Quantification of retrograde mitochondrial speed in WT and DLK KO neurons. No significant differences observed. N=3 independent differentiations, N≥ 30 axons. No significant differences observed (ns).
- **p)** Quantification of the number of mitochondria per 100 µm in WT and DLK KO axons. No significant differences observed. N=3 independent differentiations, N≥ 30 axons. No significant differences observed (ns).

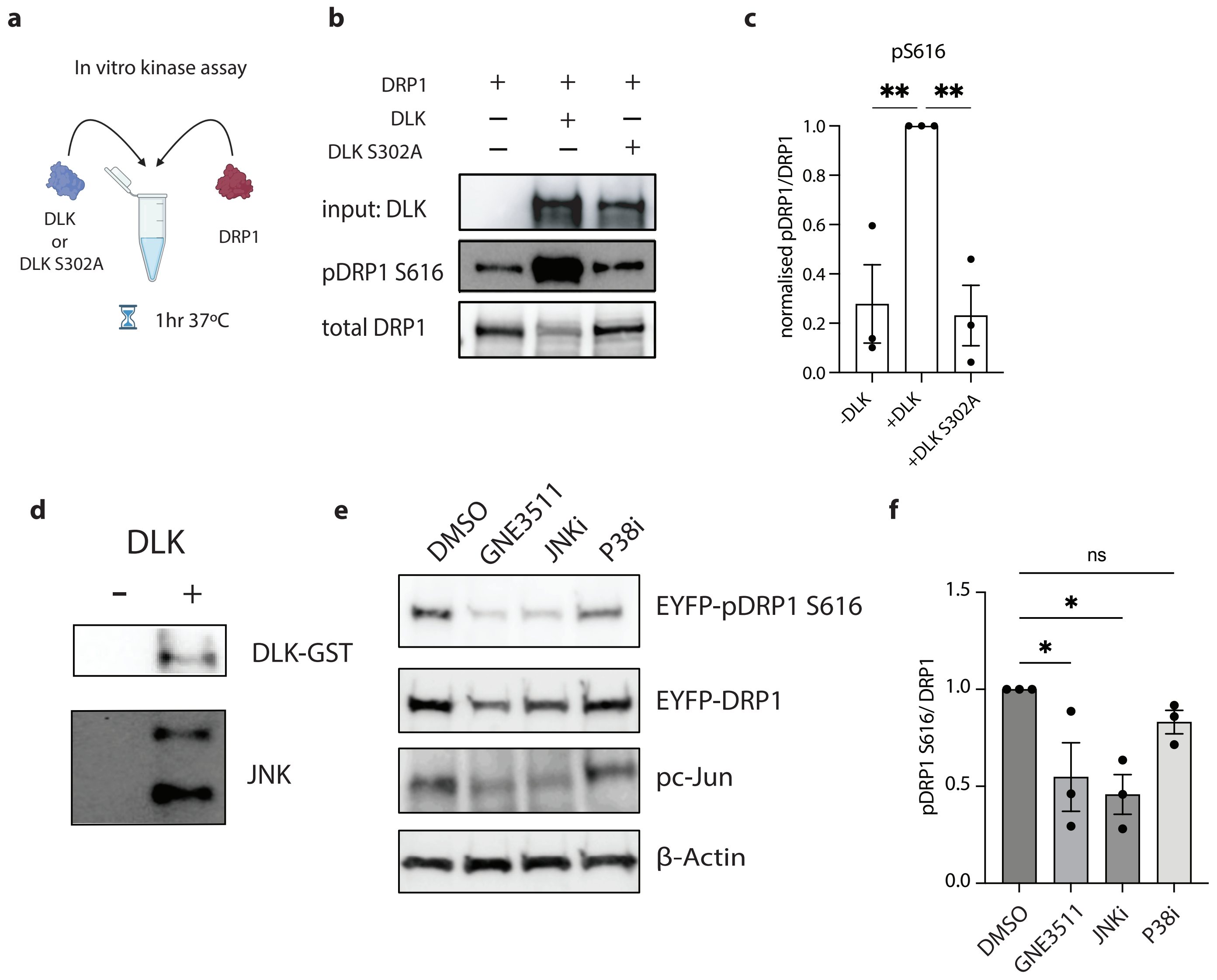
a
In vitro kinase assay
DLK
or
DLK S302A
DRP1
1hr 37ºC
b
DRP1
DLK
DLK S302A
input: DLK
pDRP1 S616
total DRP1
c
pS616
normalised pDRP1/DRP1
-DLK
+DLK
+DLK S302A
d
DLK
DLK-GST
JNK
e
DMSO
GNE3511
JNKi
P38i
EYFP-pDRP1 S616
EYFP-DRP1
pc-Jun
β-Actin
f
ns
pDRP1 S616/ DRP1
DMSO
GNE3511
JNKi
P38i

Sup Figure 3 – the DLK/JNK pathway phosphorylates DRP1 in vitro

a) Schematic representation of the *in vitro* kinase assay using purified DLK, DLK S302A and DRP1 to test the direct phosphorylation of DRP1 by DLK.
b) Representative western blots of *In vitro* kinase assay utilizing separately purified DRP1, WT DLK and kinase dead DLK S302A. Representative immunoblots for pS616-DRP1, total DRP1 and DLK
c) Quantification of Quantification of pS616-DRP1/total DRP1 in an *in vitro* kinase assay. Results are represented as mean ± SEM. One-way ANOVA, Bonferroni correction ($p \leq 0.01$ **).
d) Representative western blot of JNK co-precipitating with purified DLK-GST in HEK-293T cells. Immunoblots for total DLK and JNK.
e) Representative western blots of HEK293A cells transfected for 24 hours with DLK-GFP and EYFP-DRP1 and treated with DMSO, GNE-3511, JNKi and P38i. Immunoblot for pS616-DRP1, total DRP1, pS63-cJun and loading control ß actin.
f) Quantification of pS616-DRP1/total DRP1 levels after 24-hour expression of DLK-GFP and EYFP-DRP1 treated with DMSO, GNE-3511, JNKi and P38i. Results are represented as mean ± SEM. One-way ANOVA, Bonferroni correction (not significant (ns), $p \leq 0.05$ *).

Supplementary Figure 4

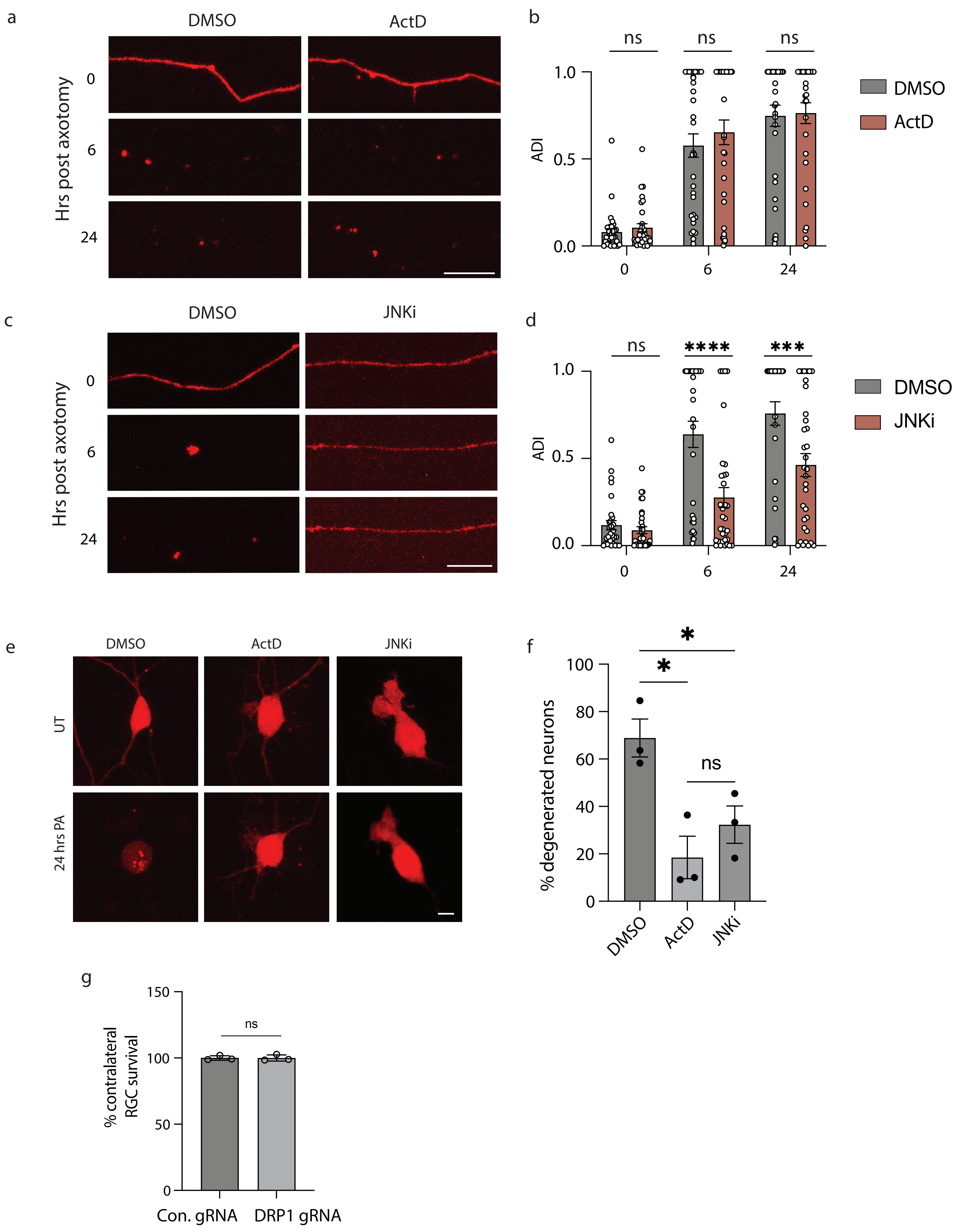

Sup Figure 4 – Effect of blocking transcription and JNK on cell death and axon degeneration after axotomy

a) Representative images of neuron axons treated with DMSO or Actinomycin D (ActD) transduced with cyto mApple (red) proximal to the site of injury 0, 6 and 24 hours post axotomy. Scale bar = 25 µm.
b) Quantification of axon degeneration index (ADI) in neurons treated with DMSO or Actinomycin D (ActD) 0, 4, 8 and 24 hours post axotomy. Results are represented as mean ± SEM. N=3 independent differentiations, N≥ 32 axons. Two-way ANOVA, Bonferroni correction (not significant, ns).
c) Representative images of neuron axons treated with DMSO or JNKi transduced with cyto mApple (red) proximal to the site of injury 0,4, 8 and 24 hours post axotomy. Scale bar = 25 µm.
d) Quantification of axon degeneration index (ADI) in neurons treated with DMSO or JNKi 0, 4, 8 and 24 hours post axotomy. Results are represented as mean ± SEM. N=3 independent differentiations, N≥ 31 axons. Two-way ANOVA, Bonferroni correction (p≤0.005 ***, p≤0.001 ****).
e) Representative images of neuron cell bodies transduced with cyto mApple (red) treated with DMSO, Actinomycin D (ActD) and JNKi pre and 24 hours post axotomy (PA). Scale bar = 40 µm.
f) Percentage degenerated neurons treated with DMSO, Actinomycin D (ActD) and JNKi 24 hours post axotomy. Results are represented as mean ± SEM. N=3 independent differentiations. Unpaired t-test (not significant, ns. p≤0.05 *).
g) Quantification of percentage RBPMS-positive RGCs in the retinas of contralateral control and DRP1 gRNA mice at 7DPI (normalized to Control gRNA). N=3 mice per condition. not significant, ns.

Uncropped western blots

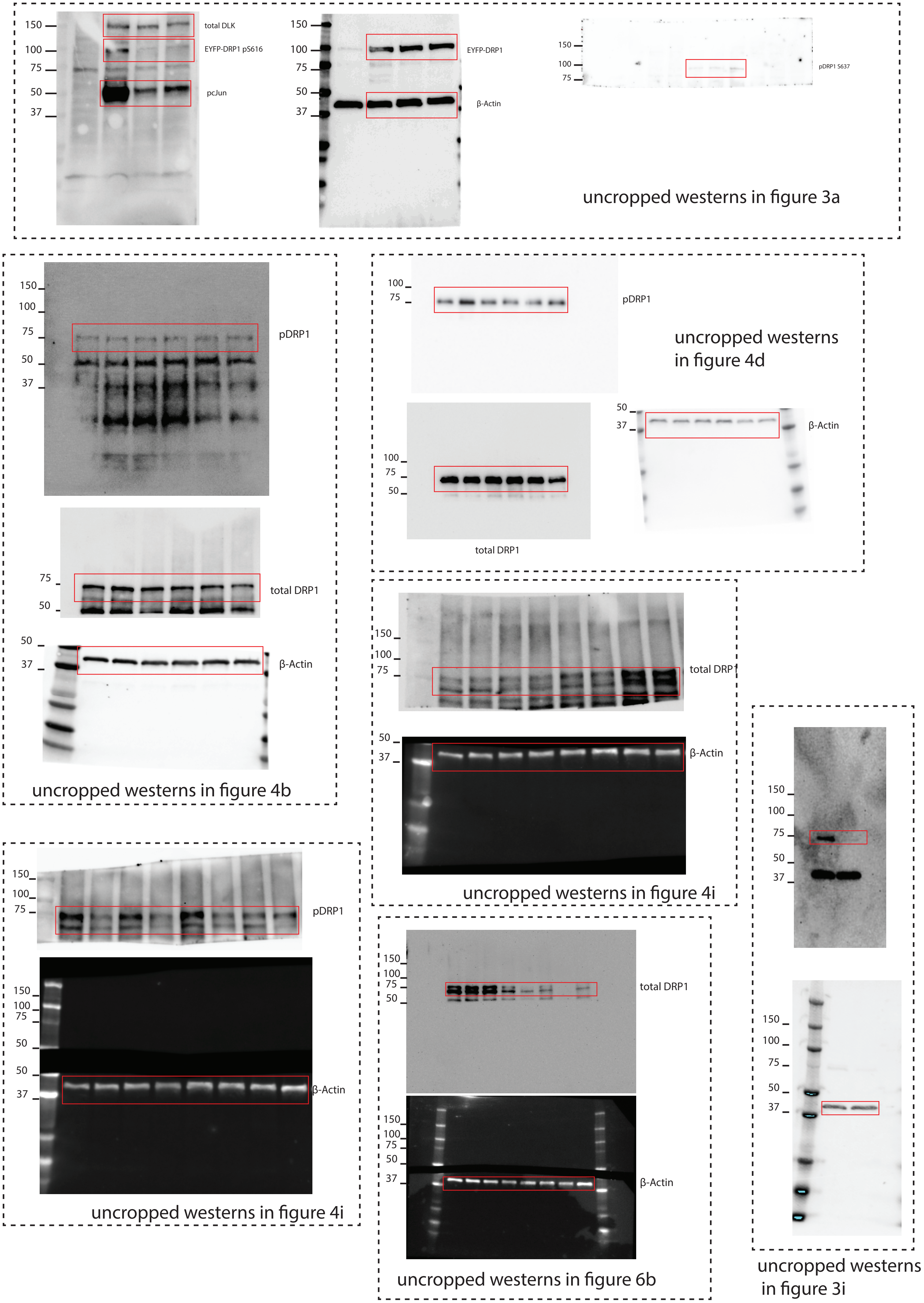

Uncropped western blots

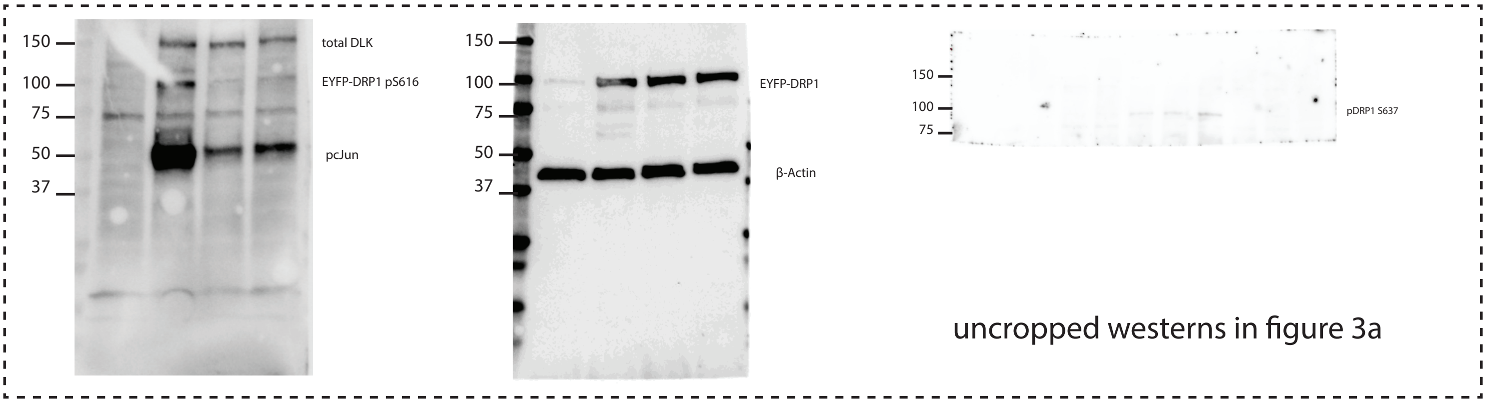

uncropped westerns in figure 3a

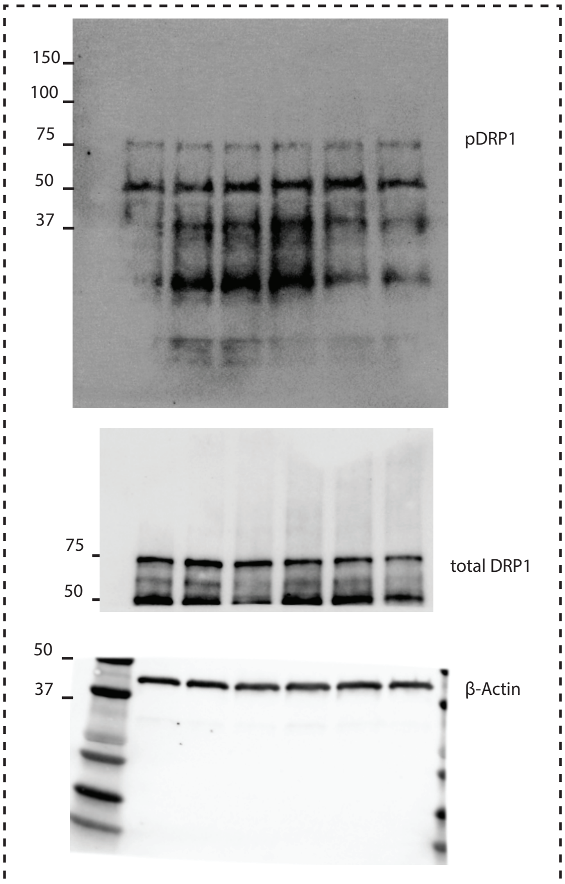

uncropped westerns in figure 4b

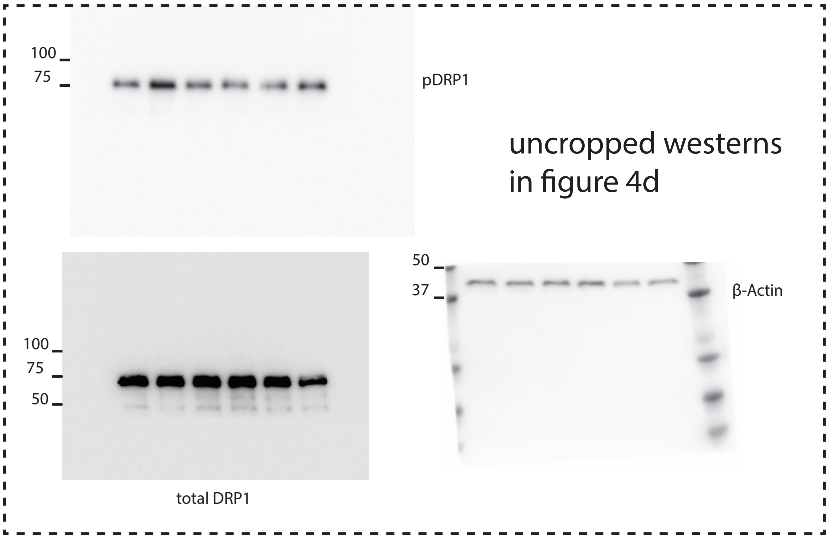

uncropped westerns in figure 4d

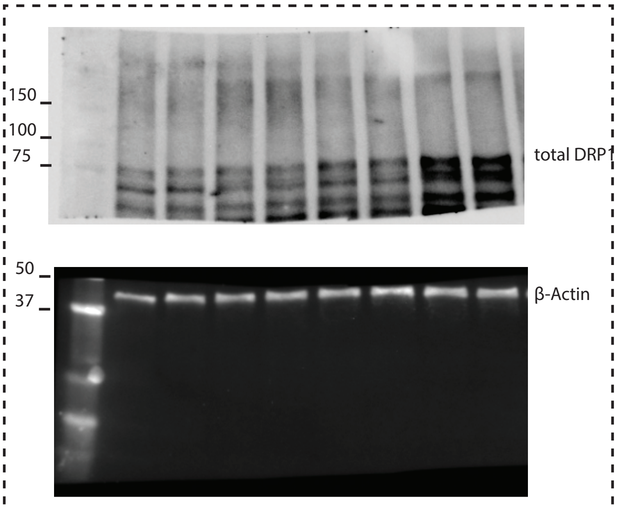

uncropped westerns in figure 4i

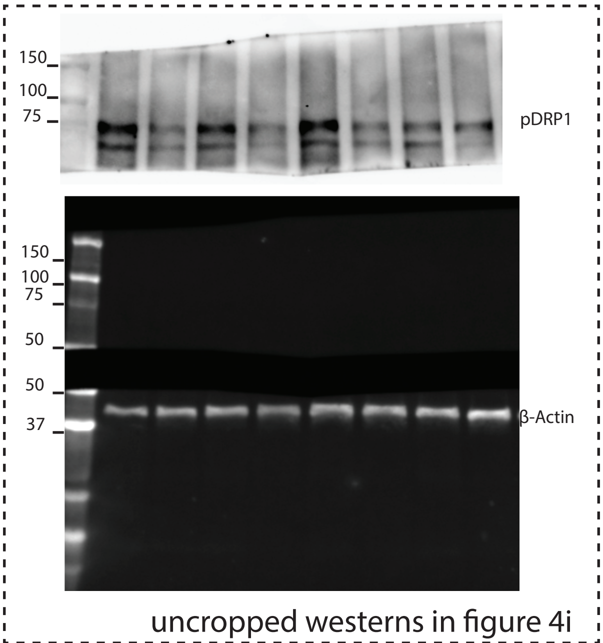

uncropped westerns in figure 4i

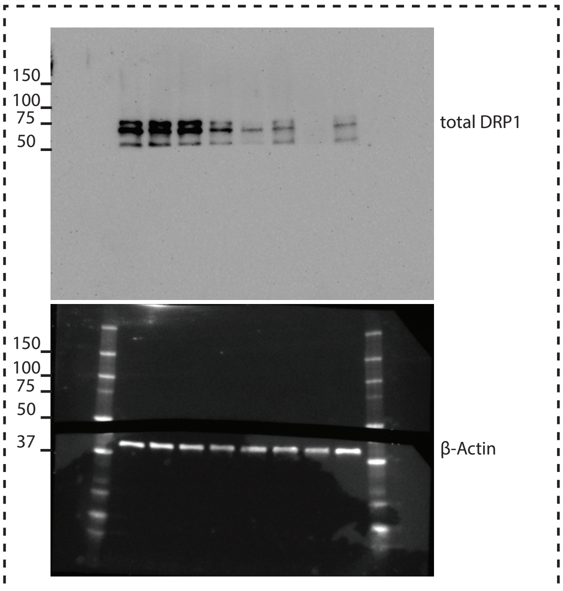

uncropped westerns in figure 6b

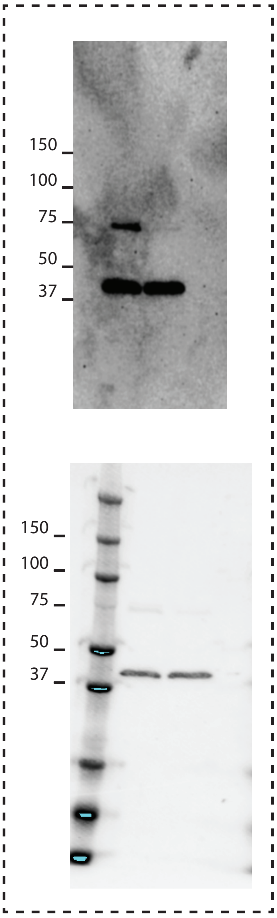

uncropped westerns in figure 3i
