# Supplementary material for: DLK-dependent axonal mitochondrial fission drives degeneration after axotomy
Source: Nat Commun. 2024 Dec 30;15:10806. doi: 10.1038/s41467-024-54982-9 (PMC11686342; doi:10.1038/s41467-024-54982-9)
Supplement: Supplementary file 2 — Description of Additional Supplementary Files [file 41467_2024_54982_MOESM2_ESM.pdf]

Description of Additional Supplementary Files

File Name: Supplementary Movie 1
Description: Mitochondrial fission after axotomy

File Name: Supplementary Movie 2
Description: Calcium influx after axotomy in DMSO-treated neurons

File Name: Supplementary Movie 3
Description: Calcium influx after axotomy in BAPTA-treated neurons

File Name: Supplementary Movie 4
Description: Mitochondrial fission after axotomy in DMSO-treated neurons

File Name: Supplementary Movie 5
Description: Mitochondrial fission after axotomy in BAPTA-treated neurons

File Name: Supplementary Movie 6
Description: Wave of mitochondrial fission moving retrogradely towards the cell body after axotomy

File Name: Supplementary Movie 7
Description: Progressive axon degeneration of the proximal axon after axotomy

File Name: Supplementary Movie 8
Description: Time lapse of neuron cell body after injury expressing cytoplasmic mAPPLE

File Name: Supplementary Movie 9
Description: Time lapse of neuron cell body after injury expressing mito-GFP

File Name: Supplementary Movie 10
Description: DLK-GFP localization at the site of mitochondrial fission after axotomy

File Name: Supplementary Movie 11
Description: Mitochondrial fission after axotomy in WT neurons

File Name: Supplementary Movie 12
Description: Mitochondrial fission after axotomy in DLK KO neurons

File Name: Supplementary Movie 13
Description: Mitochondrial fission after axotomy in DMSO-treated neurons

File Name: Supplementary Movie 14
Description: Mitochondrial fission after axotomy in GNE 3511-treated neurons

File Name: Supplementary Movie 15
Description: Mitochondrial fission after axotomy in DLK KO neurons expressing DLK-GFP

File Name: Supplementary Movie 16
Description: Mitochondrial fission after axotomy in DLK KO neurons expressing DLK-C127S-GFP

File Name: Supplementary Movie 17
Description: Mitochondrial fission after axotomy in DLK KO neurons expressing DLK-S302A-GFP
