# Supplementary material for: DLK-dependent axonal mitochondrial fission drives degeneration after axotomy
Source: Nat Commun. 2024 Dec 30;15:10806. doi: 10.1038/s41467-024-54982-9 (PMC11686342; doi:10.1038/s41467-024-54982-9)
Supplement: Supplementary file 20 — Reporting Summary [file 41467_2024_54982_MOESM20_ESM.pdf]

nature portfolio

Corresponding author(s): Dr. Claire Le Pichon

Last updated by author(s): Nov 1, 2024

Reporting Summary

Nature Portfolio wishes to improve the reproducibility of the work that we publish. This form provides structure for consistency and transparency in reporting. For further information on Nature Portfolio policies, see our Editorial Policies and the Editorial Policy Checklist.

Statistics

For all statistical analyses, confirm that the following items are present in the figure legend, table legend, main text, or Methods section.

n/a	Confirmed	
☐	☒	The exact sample size (*n*) for each experimental group/condition, given as a discrete number and unit of measurement
☐	☒	A statement on whether measurements were taken from distinct samples or whether the same sample was measured repeatedly
☐	☒	The statistical test(s) used AND whether they are one- or two-sided<br>*Only common tests should be described solely by name; describe more complex techniques in the Methods section.*
☐	☒	A description of all covariates tested
☐	☒	A description of any assumptions or corrections, such as tests of normality and adjustment for multiple comparisons
☐	☒	A full description of the statistical parameters including central tendency (e.g. means) or other basic estimates (e.g. regression coefficient) AND variation (e.g. standard deviation) or associated estimates of uncertainty (e.g. confidence intervals)
☒	☐	For null hypothesis testing, the test statistic (e.g. *F*, *t*, *r*) with confidence intervals, effect sizes, degrees of freedom and *P* value noted<br>*Give P values as exact values whenever suitable.*
☒	☐	For Bayesian analysis, information on the choice of priors and Markov chain Monte Carlo settings
☒	☐	For hierarchical and complex designs, identification of the appropriate level for tests and full reporting of outcomes
☒	☐	Estimates of effect sizes (e.g. Cohen's *d*, Pearson's *r*), indicating how they were calculated

Our web collection on statistics for biologists contains articles on many of the points above.

Software and code

Policy information about availability of computer code

Data collection	N/A
Data analysis	Graphpad prism was used for the statistical analysis. ImageJ was used for western blot and image analysis.

For manuscripts utilizing custom algorithms or software that are central to the research but not yet described in published literature, software must be made available to editors and reviewers. We strongly encourage code deposition in a community repository (e.g. GitHub). See the Nature Portfolio guidelines for submitting code & software for further information.

Data

Policy information about availability of data

All manuscripts must include a data availability statement. This statement should provide the following information, where applicable:

- Accession codes, unique identifiers, or web links for publicly available datasets
- A description of any restrictions on data availability
- For clinical datasets or third party data, please ensure that the statement adheres to our policy

N/A

Human research participants

Policy information about studies involving human research participants and Sex and Gender in Research.

Reporting on sex and gender	N/A
Population characteristics	N/A
Recruitment	N/A
Ethics oversight	N/A

Note that full information on the approval of the study protocol must also be provided in the manuscript.

Field-specific reporting

Please select the one below that is the best fit for your research. If you are not sure, read the appropriate sections before making your selection.

[x] Life sciences [ ] Behavioural & social sciences [ ] Ecological, evolutionary & environmental sciences

For a reference copy of the document with all sections, see nature.com/documents/nr-reporting-summary-flat.pdf

Life sciences study design

All studies must disclose on these points even when the disclosure is negative.

Sample size	No statistical methods were used to determine sample sizes; these were based on prior experiments by the authors and in the literature.
Data exclusions	no data was excluded
Replication	In vitro experiments were replicated at least 3 times through independent neuronal differentiations
Randomization	N/A
Blinding	Quantification was done in a blinded manner

Reporting for specific materials, systems and methods

We require information from authors about some types of materials, experimental systems and methods used in many studies. Here, indicate whether each material, system or method listed is relevant to your study. If you are not sure if a list item applies to your research, read the appropriate section before selecting a response.

Materials & experimental systems

n/a	Involved in the study	
[ ]	[x]	Antibodies
[ ]	[x]	Eukaryotic cell lines
[x]	[ ]	Palaeontology and archaeology
[ ]	[x]	Animals and other organisms
[x]	[ ]	Clinical data
[x]	[ ]	Dual use research of concern

Methods

n/a	Involved in the study	
[x]	[ ]	ChIP-seq
[x]	[ ]	Flow cytometry
[x]	[ ]	MRI-based neuroimaging

Antibodies

Antibodies used	The following antibodies were used at the concentrations stated: anti-DRP1 (Santa Cruz Biotechnology, cat. no. sc-101270, 1:1000), anti-pDRP1 S616 (CST, cat. no. 3455S, 1:1000), anti-ß Actin (Sigma, cat. no. A1978, 1:5000), anti-p-cJun S63 (CST, cat. no. 9261, 1:1000), anti-pDRP1 S637 (CST, cat. no. 4867S, 1:1000). DLK antibody developed and kindly provided by Cell Signaling Technology, clone cat. no. GF-23-1, 1:500)
Validation	Each antibody was validated by the manufacturer except for anti-DLK which was validated in our lab using DLK KO cells (Fig S2H) .

Eukaryotic cell lines

Policy information about cell lines and Sex and Gender in Research

Cell line source(s)	HEK 293A cells were a gift from Dr. Tamas Balla's Lab at NICHD. i3N iPSCs (from male donor) were gifted by Dr. Michael Ward at NINDS.
Authentication	Cell authentication was not performed.
Mycoplasma contamination	Mycoplasma free cell culture was assessed by the absence of DAPI staining in the cytoplasm of cells
Commonly misidentified lines (See ICLAC register)	these cells are not in the database

Animals and other research organisms

Policy information about studies involving animals; ARRIVE guidelines recommended for reporting animal research, and Sex and Gender in Research

Laboratory animals	C57BL/6J mice were used for all in vivo studies
Wild animals	no wild animals were used
Reporting on sex	Equivalent numbers of male and female mice were used in experiments; no sex effects were observed.
Field-collected samples	na
Ethics oversight	Animal care and experimental procedures were performed in accordance with NICHD protocols 20-003 and 23-003 (Le Pichon lab) approved by the Eunice Kennedy Shriver National Institute of Child Health and Human Development ACUC, animal protocol number NEI-606 (Li lab) approved by the National Eye Institute ACUC, and animal protocol AN-7208 (Watkins Lab) approved by the Baylor College of Medicine ACUC.

Note that full information on the approval of the study protocol must also be provided in the manuscript.
